# Prediction value of heart rate combined with arteriosclerosis index for left coronary artery lesion in patients with ACS

**DOI:** 10.1186/s12872-025-05063-2

**Published:** 2025-08-02

**Authors:** Bike Bie, Dezhong Yang

**Affiliations:** 1 Daping Hospital, The Third Military Medical University (Army Medical University), 10th Changjiangzhilu Road, Yuzhong District, Chongqing, 400042 People’s Republic of China; 2 Department of Cardiology, Daping Hospital, The Third Military Medical University (Army Medical University), 10th Changjiangzhilu Road, Yuzhong District, Chongqing, 400042 People’s Republic of China

**Keywords:** Heart rate, Arteriosclerosis index, Acute coronary syndromes, Left coronary artery

## Abstract

**Objective:**

This study aims to evaluate the predictive value of heart rate combined with arteriosclerosis index on the degree of left coronary artery(LCA) lesions in patients with acute coronary syndromes(ACS).

**Methods:**

Patients diagnosed with ACS who were discharged from the Department of Cardiovascular Medicine of the Army Characteristic Medical Center from 2019 to 2024 were included, and the relevant clinical data of the selected patients were collected through the electronic medical record database system. According to the results of coronary angiography, they were divided into mild lesion group (All lesions in the left coronary artery (including its branches) with a stenosis degree less than 70%, *n* = 121) and severe lesion group (any one or more vessels (including their branches) in the left coronary artery with a stenosis degree of 70% or more was the severe lesion group, *n* = 264).

**Results:**

In ACS patients with concomitant LCA and right coronary artery (RCA) lesions, the results of Spearman correlation analysis indicated that heart rate (*r* = 0.1948, *P* = 0.0001) and arteriosclerosis index (*r* = 0.1636, *P* = 0.0013) were positively correlated with the Gensini score of the LCA in coronary angiography. The results of multivariate logistic regression analysis suggested that heart rate (HR:1.062, 95%CI:1.040–1.083, *P* < 0.001), arteriosclerosis index (HR:1333, 95%CI:1.045–1.699, *P* = 0.021), and age (HR:1.023, 95%CI:1.003–1.042, *P* = 0.021) were all independent predictors of severe lesions in the LCA. The ROC curve showed that heart rate(AUC = 0.70) and arteriosclerosis index(AUC = 0.59) had certain predictive value for the degree of LCA lesions, and the predictive value of combined assessment was higher (AUC = 0.72). In ACS patients with isolated LCA lesions, both HR (*r* = 0.0299, *P* = 0.7424) and AI (*r* = 0.1930, *P* = 0.0325) remained positively correlated with the severity of LCA lesions. The results of multivariate logistic regression analysis suggested that heart rate (HR:1.044, 95%CI:1.004–1.085, *P* = 0.032) and arteriosclerosis index (HR:1.72, 95%CI:1.001–2.955, *P* = 0.049) were all independent predictors of severe lesions in the LCA. The ROC curve showed that heart rate(AUC = 0.64) and arteriosclerosis index(AUC = 0.64) had certain predictive value for the degree of LCA lesions, and the predictive value of combined assessment was higher (AUC = 0.71).

**Conclusions:**

The combination of HR and AI demonstrates predictive value for LCA lesion severity in ACS patients.

**Supplementary Information:**

The online version contains supplementary material available at 10.1186/s12872-025-05063-2.

## Introduction

ACS are defined by a sudden reduction in blood supply to the heart [1]. Risk factors associated with ACS include older age, current tobacco use, diabetes, and elevated levels of lipids, blood pressure, and body mass index [2].ACS is common among older people. About 85% of deaths associated with ACS occur in those 65 years or older [3]. Studies have shown that rates of ACS have increased in younger people, between 1995 and 2014, the percentage of acute MI admissions for patients aged 35 to 54 years increased from 27–32% [4, 5]. The left coronary artery usually supplies the left atrium, most of the ventricular septum, and the left ventricle (septum, anterior wall, lateral wall), so stenosis of the left coronary artery has a greater impact on the pump function and peripheral hemodynamics of the heart [6]. Therefore, the early identification and prediction of left coronary artery lesions in ACS patients are of great significance for their prognosis. In recent years, it has been confirmed that heart rate(HR) is an important factor affecting the prognosis of cardiovascular diseases, and in the study of patients with hypertension [7], heart failure [8], and coronary heart disease [9].

Arteriosclerosis index (AI) is an index calculated from total cholesterol (TC) and high density lipoprotein-cholesterol (HDL-C)(AI=(TC-HDL-C)/HDL-C), which reflects lipid metabolism disorders and has a good positive correlation with the degree of arteriosclerosis in the human body. The calculation formula of AI is actually the mathematical evolution of the ratio of TC to HDL-C minus 1, and its clinical significance is highly consistent with the ratio of TC to HDL-C. This form may have been proposed to more intuitively reflect the ratio of non-HDL-C (atherogenic lipoprotein) to HDL-C (anti-atherogenic lipoprotein). The Framingham Heart Study in the United States found that the ratio of TC to HDL-C was a better predictor of cardiovascular disease risk than total cholesterol (TC) or low-density lipoprotein cholesterol (LDL-C) alone when analyzing blood lipids and the risk of coronary heart disease [10]. In many studies on blood lipids and atherosclerosis risk, similar formulas have been used to quantify lipid abnormalities. For example, Japanese scholar Ueshima H used this formula to assess the risk of arteriosclerosis in the study of metabolic syndrome [11], and the 4 S trial (Scandinavian Simvastatin Survival Study) also used the ratio of non-HDL-C to HDL-C to evaluate the efficacy of statins [12]. The National Cholesterol Education Program (NCEP ATP III, 2001) in the United States officially included non-HDL-C (Non-HDL-C = TC - HDL-C) in the lipid management guidelines as a secondary treatment target, further promoting the use of similar formulas [13, 14].

Most of the previous studies have focused on the plasma AI on coronary heart disease and the degree of coronary artery disease, and most of the studies on the degree of coronary artery disease have focused on the whole of the three major coronary arteries, and there are few independent studies on the LCA and the RCA. The aim of this study was to investigate the predictive value of HR and AI in the degree of isolated left coronary artery disease. HR and lipid profiles obtained during initial hospitalization are readily accessible clinical parameters for ACS patients. This study aims to utilize these simple and rapidly available indicators for early prognosis prediction, thereby enabling clinicians to provide more comprehensive and precise therapeutic interventions during the critical early stages of ACS.

## Methods

### Research subjects and groups

**Fig. 1:**
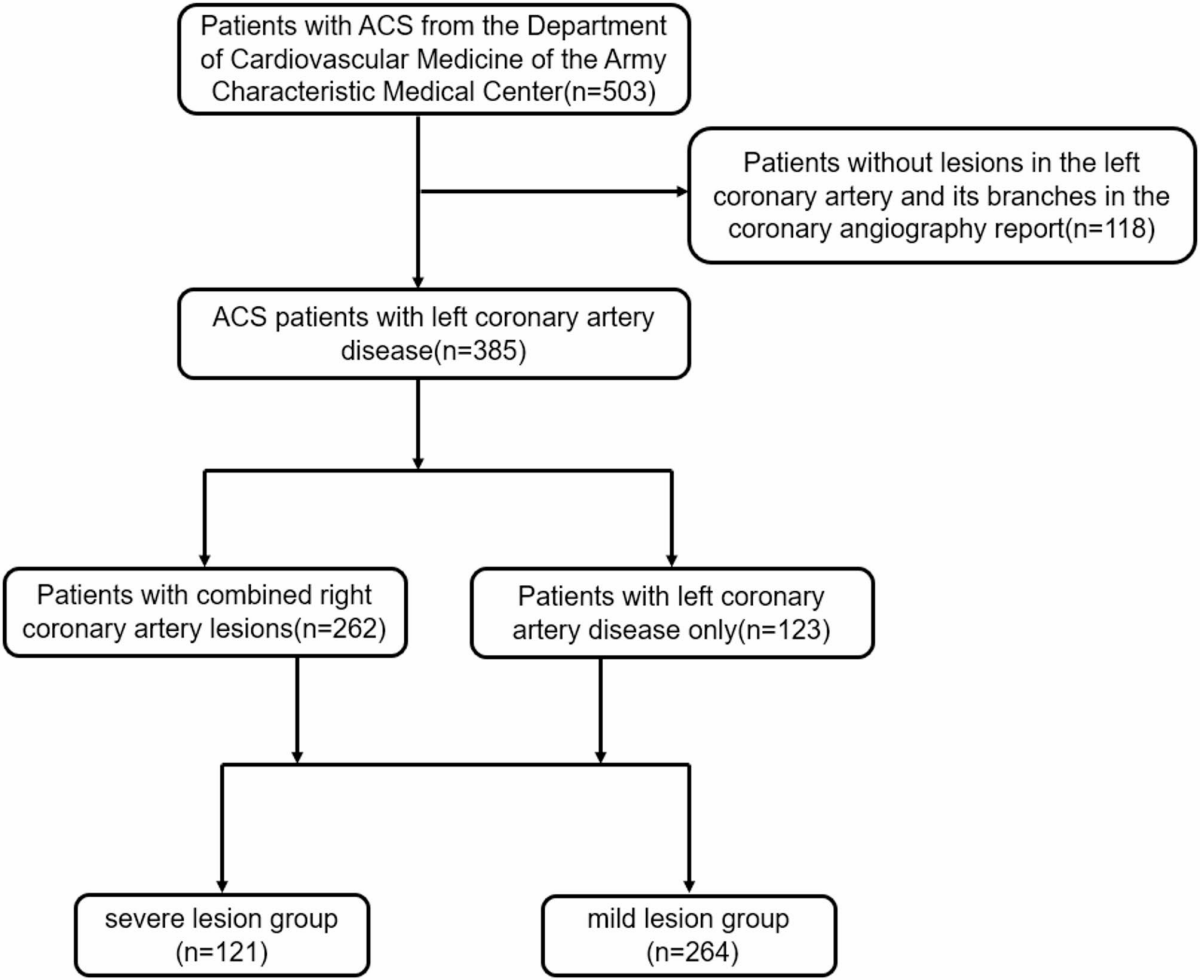
Flowchart of participant inclusion criteria

This study is a retrospective study. A total of 385 patients diagnosed with ACS in the Department of Cardiovascular Medicine of the Army Characteristic Medical Center from March 2019 to June 2024 were enrolled. The cohort included 262 patients with multivessel lesions (involving both LCA and RCA) and 123 patients with single-vessel LCA lesions. Braunwald’s Heart Disease (12th Edition, 2022) points out that coronary artery stenosis of ≥ 70% is generally considered “hemodynamically significant”, which may lead to myocardial ischemia, especially during exercise or stress [15]. The FAME study (NEJM, 2009) confirmed through the fractional flow reserve (FFR) that FFR ≤ 0.80 (corresponding to approximately 70–75% stenosis) is the threshold for functional ischemia, guiding whether revascularization is needed [16]. Therefore, we use 70% coronary artery stenosis to define severe and mild lesions. And they were grouped according to the degree of LCA lesions in the reports of coronary angiography, the stenosis of at least one blood vessel in the LCA(including its branches) ≥ 70% in the severe lesion group (*n* = 264), and all lesions in the LCA (including its branches) with a stenosis degree less than 70% in the mild lesion group (*n* = 121).(The Graphical abstract shown in Fig. 1).

### Inclusion criteria and exclusion criteria

#### Inclusion criteria

(1) Have coronary angiography reports and meet the diagnostic criteria for ACS(Including unstable angina, ST-segment elevation myocardial infarction (STEMI) and non-ST-segment elevation myocardial infarction(NSTEMI)); (2) Age ≥ 18 years old; (3) There is a stenosis lesion in the LCA(With or without RCA lesions).

#### Exclusion criteria

(1) Patients without coronary angiography reports; (2) Patients without stenosis lesions in the LCA; (3) Patients with incomplete clinical data; (4) Prior history of coronary artery bypass grafting or percutaneous coronary intervention; (5) History of major trauma or surgical procedures within 3 months prior to admission; (6) Combined with valvular heart disease, rheumatic heart disease, cardiomyopathy; (7) Combined with malignant tumors or other serious diseases.

### Collection of general clinical data

Demographic and clinical characteristics were obtained through comprehensive review of the electronic medical record database. It mainly includes gender, age, body mass index (BMI), systolic blood pressure (SBP), diastolic blood pressure (DBP), heart rate (HR), comorbidities: atrial fibrillation (AF), hypertension, diabetes, valvular heart disease, Chronic kidney disease (CKD) (eGFR < 60 ml/min/1.73m^2^), TC, Triglyceride (TG), HDL-C, Low density lipoprotein-cholesterol (LDL-C), AI=(TC-HDL-C)/HDL-C, Gensini score [17] etc. All Gensini scores were assessed by experienced cardiologists in our catheterization laboratory according to the pre-specified criteria from the original literature [18].

### Establishment and evaluation of the predictive model

The ACS patients was randomly split into training (80%) and test (20%) sets. Tenfold crossvalidation was performed in each model to prevent overfitting to acquire average accuracy. The performance of each model was evaluated by the Area Under the Curve(AUC) and average precision (AP) from precision-recall curves in the validation set.

### Statistical analyses

The normally distributed continuous data were represented by the mean ± standard deviation (X ± S), and the non-normally distributed continuous data were expressed as M(Q1,Q3), and Mann-Whitney U was used for comparison between groups. The count data were expressed in frequency (%), and the Chi-square test was used for comparison between groups. For correlation analysis, Pearson correlation analysis is used if the data is normally distributed, otherwise Spearman correlation analysis is used. The receiver operating characteristic curve (ROC) was employed to assess the predictive value of AI and HR for LCA lesion severity in ACS patients, and the ROC curves of HR, *P* < 0.05 indicates that the difference is statistically significant. AUC values of 0.8–0.9 indicate excellent discrimination, 0.7–0.8 acceptable discrimination, and 0.5–0.7 poor discrimination. An AUC of 0.5 suggests no discriminative ability [19]. Spearman’s ρ ranges from − 1 to + 1. A value of + 1 means a perfect increasing monotonic relationship, − 1 a perfect decreasing relationship, and 0 no monotonic correlation. Values between 0.7 and 1.0 (− 0.7 to − 1.0) suggest strong monotonic association [20].

## Results

### Clinical baseline data for patients with ACS

A total of 385 patients with ACS were enrolled in this study, including 121 (31.43%) in the mild lesion group and 264 (68.57%) in the severe lesion group. There were no statistically significant differences between the two groups in terms of gender, age, BMI, systolic blood pressure, diastolic blood pressure, smoking, HDL-C, beta blockers, history of atrial fibrillation, history of hypertension, history of diabetes mellitus, history of CKD, and valvular heart disease (*P* > 0.05) (Table 1).

**Table 1:** Clinical baseline data for patients with ACS

Variable	Mild lesion group (*N* = 121)	Severe lesion group (*N* = 264)	*P*-value
Female sex, no. (%)	24 (19.84)	60 (22.73)	0.524
Age, year, M (Q₁, Q₃)	64 (54, 69)	64 (55, 74)	0.495
BMI, kg/m2, M (Q₁, Q₃)	24.24 (22.49, 26.12)	24.24 (22.04, 26.57)	0.870
SBP, mmHg, M (Q₁, Q₃)	130 (113, 140)	127 (113, 141)	0.847
DBP, mmHg, M (Q₁, Q₃)	75 (65, 83)	78(69, 85)	0.105
HR, beats/min, M (Q₁, Q₃)	70 (63, 79)	80 (71, 90)	< 0.001
Smoking status, no.(%)			0.165
Never	49 (40.50)	118 (44.70)	
Former	59 (48.76)	104 (39.94)	
Current	13 (10.74)	42 (15.91)	
TC(mmol/l), M (Q₁, Q₃)	4.45 (3.81, 4.92)	4.71 (4.18, 5.29)	0.003
TG(mmol/l), M (Q₁, Q₃)	1.44 (0.93,1.93)	1.72 (1.14, 2.18)	0.016
HDL-C(mmol/l), M (Q₁, Q₃)	1.10 (0.90, 1.22)	1.11 (0.93, 1.21)	0.688
LDL-C(mmol/l), M (Q₁, Q₃)	2.75 (2.19, 3.04)	2.88 (2.55, 3.35)	0.003
AI, M (Q₁, Q₃)	3.45 (2.81, 3.92)	3.71 (3.18, 4.29)	0.003
Medication in hospital, n (%)			
Beta blockers	78 (64.46)	175 (66.29)	0.815
Aspirin/Indobufen	117 (96.694)	260 (98.485)	0.266
Clopidogrel/Ticagrelor	117 (96.694)	261 (98.864)	0.213
Statins	119(98.35)	260(98.49)	0.919
TIMI flow(cuprit vessel)			0.059
0	50 (58.14)	134 (53.82)	
1	11 (12.791)	14 (5.62)	
2	8 (9.30)	24 (9.64)	
3	17 (19.77)	77 (30.92)	
Three-vessel disease	9 (7.44)	48 (18.18)	0.009
Comorbidities, no(%)			
Atrial fibrillation	6 (4.96)	21 (7.96)	0.285
Hypertension	76 (66.67)	143 (57.43)	0.095
Diabetes	32 (26.45)	80 (30.30)	0.598
CKD	10 (9.09)	25 (9.47)	0.146
Valvulopathy	3 (2.48)	4 (1.52)	0.511
ACS type, no. (%)			
Unstable angina	9 (7.44)	2 (0.76)	0.001
NSTEMI	38 (31.41)	67 (25.38)	0.267
STEMI	74 (61.16)	195 (73.86)	0.016
Gensini score (LCA), M (Q₁, Q₃)	16 (7, 30)	53 (40, 82)	< 0.001
Lesions of RCA, no.(%)	93 (76.86)	169 (64.02)	0.017

BMI: body mass index; SBP: systolic blood pressure; DBP: diastolic blood pressure; HR: heart rate; TC: total cholesterol; TG:triglyceride; HDL-C: high density lipoprotein-cholesterol; LDL-C low-density lipoprotein cholesterol; AI: arteriosclerosis index; ACS: Acute coronary syndromes; NSTEMI: non-ST-segment elevation myocardial infarction; STEMI: ST-segment elevation myocardial infarction; CKD: chronic kidney disease; LCA: left coronary artery; RCA: right coronary artery

### Correlation analysis between HR, AI and left coronary gensini score

**Fig. 2:**
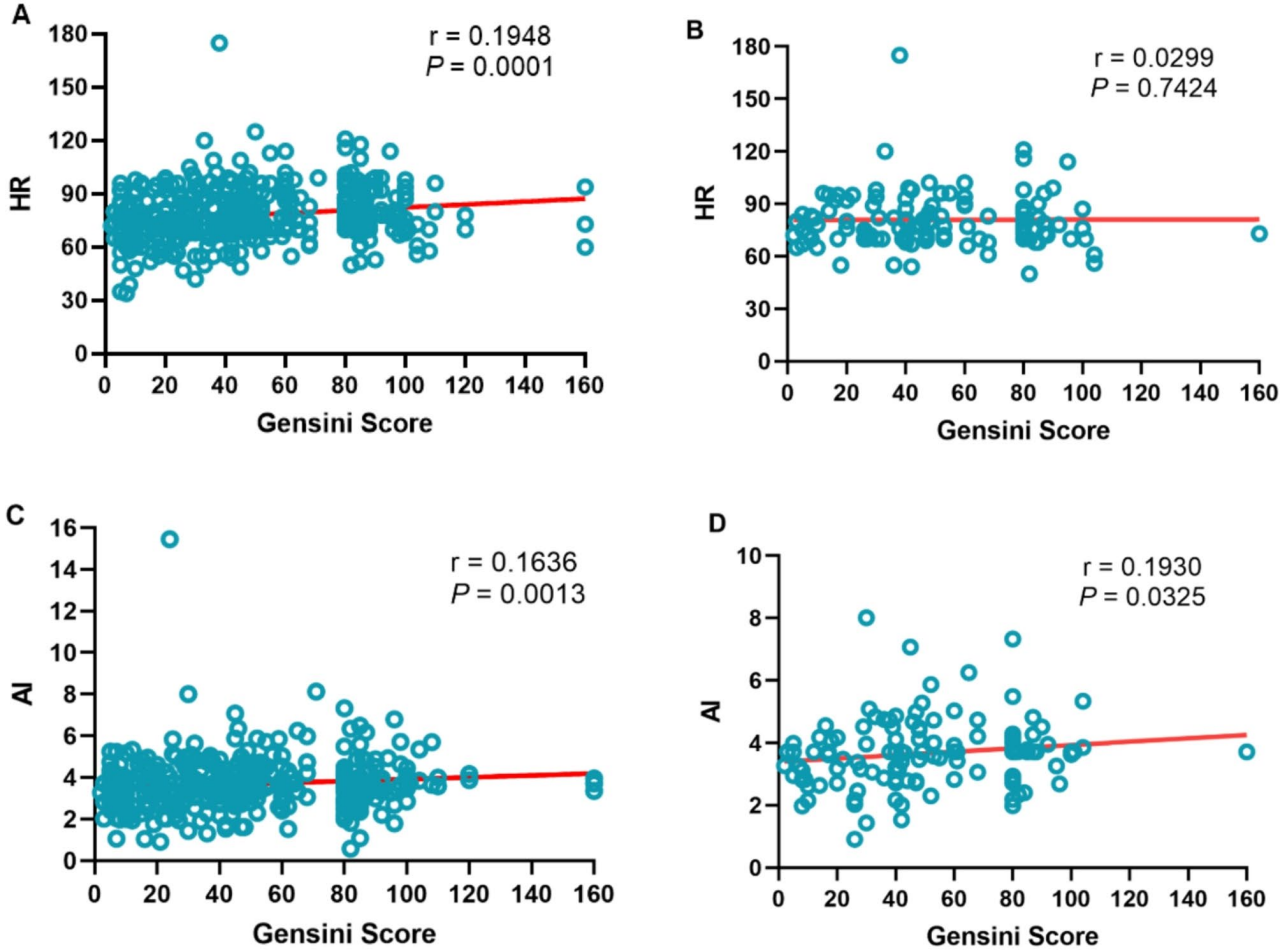
Scatter plot of the correlation between HR, AI and left coronary Gensini score. **A**, **C**: LCA lesions (with or without RCA lesions); **B**, **D**: LCA lesions(without RCA lesions). HR: heart rate; AI: arteriosclerosis index

Spearman correlation analysis was performed between research indicators such as HR and AI and left coronary Gensini score. The results of Spearman correlation analysis showed that HR (*r* = 0.1948, *P* = 0.0001) and AI (*r* = 0.1636, *P* = 0.0013) were positively correlated with the Gensini score of the LCA in ACS patients with concomitant RCA lesions (Fig. 2 A, C). In ACS patients with isolated LCA lesions, both HR (*r* = 0.0299, *P* = 0.7424) and AI (*r* = 0.1930, *P* = 0.0325) remained positively correlated with the severity of LCA lesions (Fig. 2B, D).

### Univariate and multivariate logistic regression analysis

Univariate and multivariate logistic regression analyses were performed to determine the predictors of severe coronary artery stenosis in patients with ACS. Multivariate logistic regression analysis was performed for variables with a *P* < 0.05 in univariate logistic regression analysis. Multivariate logistic regression analysis showed that HR and AI were independent predictors of severe LCA lesions(With or without RCA lesions) in ACS patients (Table 2).

**Table 2:** Results of logistic regression analysis of severe coronary artery lesions in ACS patients

Variable	LCA lesions(with or without RCA lesions)				LCA lesions (without RCA lesions)			
	Unadjusted OR (95% CI)	*P* value	Adjusted OR (95% CI)	*P* value	Unadjusted OR (95% CI)	*P* value	Adjusted OR (95% CI)	*P* value
Female	1.189(0.699–2.023)	0.524			0.599(0.237—1.518)	0.28		
Age	1.007(0.991–1.022)	0.412	1.023(1.003–1.042)	0.021	1.009(0.98—1.039)	0.541	1.03(0.994–1.067)	0.105
BMI	0.995(0.934–1.060)	0.873			0.958(0.847–1.084)	0.495		
SBP	1.001(0.992–1.011)	0.815			0.997(0.98—1.015)	0.775		
DBP	1.014(0.999–1.029)	0.068			1.002(0.974—1.031)	0.889		
HR	1.061(1.041–1.081)	< 0.001	1.062(1.040–1.083)	< 0.001	1.047(1.007—1.088)	0.02	1.044(1.004–1.085)	0.032
TC	1.379(1.111–1.712)	0.004			1.52(0.975—2.367)	0.064		
TG	1.246(1.003–1.548)	0.047	1.173(0.918–1.499)	0.203	1.211(0.831—1.764)	0.32	1.005(0.643–1.569)	0.984
HDL-C	0.777(0.406–1.489)	0.447			0.708(0.13—3.858)	0.69	0.324(0.036–2.926)	0.315
Smoking status	0.932(0.604–1.439)	0.752	1.154(0.696–1.915)	0.579	1.2(0.513—2.807)	0.674	1.216(0.463–3.197)	0.692
Hypertension	0.675(0.424–1.072)	0.096			0.478(0.18—1.274)	0.14		
Beta blockers	1.084 (0.690–1.702)	0.726	0.846 (0.509–1.405)	0.518	2.1(0.874—5.047)	0.097	1.684(0.638–4.443)	0.293
AI	1.379(1.111–1.712)	0.004	1.333(1.045–1.699)	0.021	1.52(0.975–2.367)	0.064	1.72(1.001–2.955)	0.049
Lesions of RCA	0.536(0.328–0.876)	0.013	0.604(0.355–1.028)	0.063	–	–	–	–

BMI: body mass index; SBP: systolic blood pressure; DBP: diastolic blood pressure; HR: heart rate; TC: total cholesterol; TG:triglyceride; AI: arteriosclerosis index; CI: confidence interval; OR: odds ratio; RCA: right coronary artery; LCA: left coronary artery

### HR, AI, and a combination of the two to predict the ROC curve of severe LCA lesions in patients with ACS

**Fig. 3:**
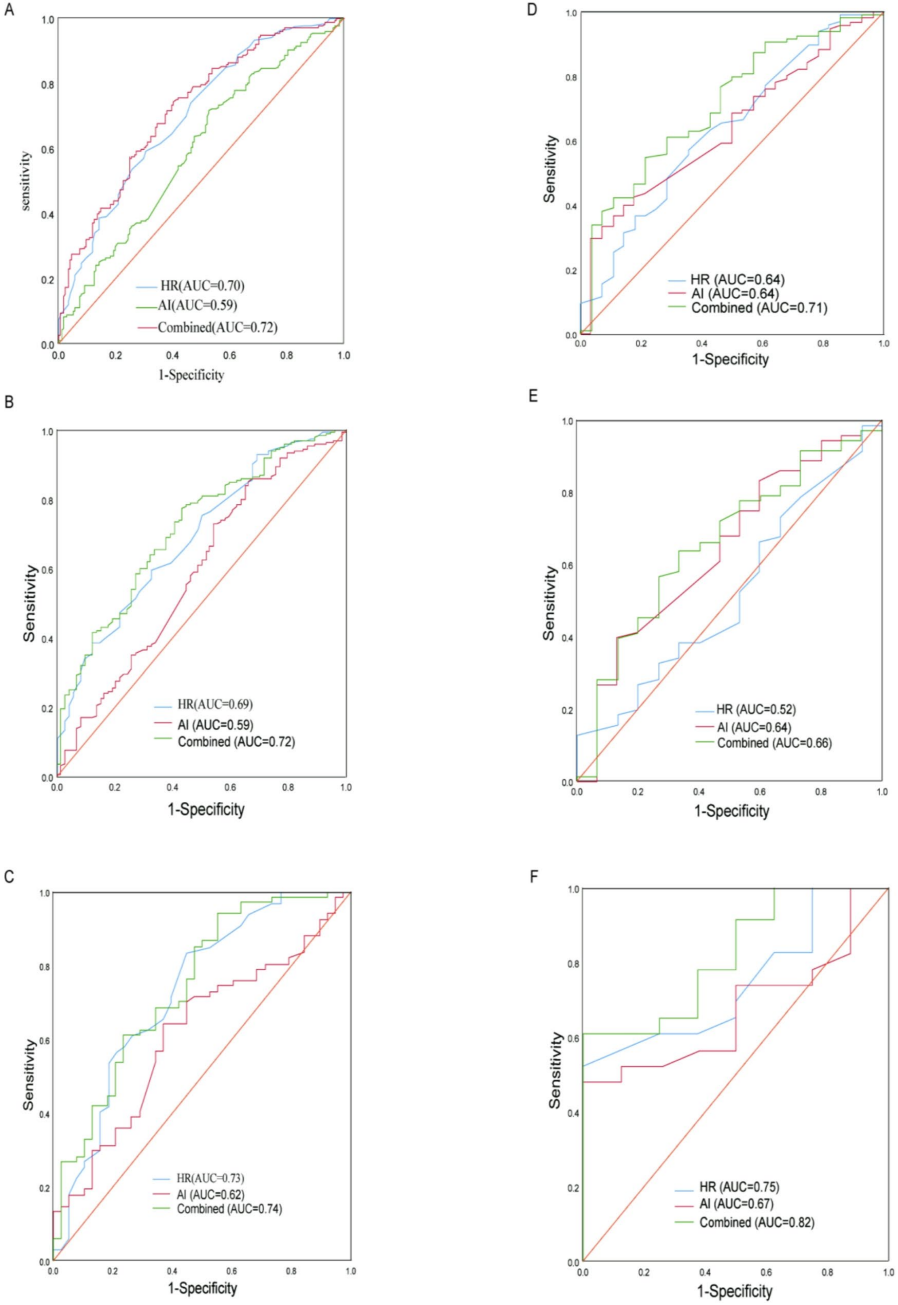
LCA lesions(with or without RCA lesions): ROC curves of ACS: (**A**)HR(95%CI: 0.644–0.756, *P* < 0.001), ROC curves of STEMI: (**B**)AI(95%CI: 0.533–0.656, *P* = 0.003), ROC curves of NSTEMI: (**C**)combined(95%CI: 0.667–0.775, *P* < 0.001). LCA(without RCA lesions): ROC curves of ACS: (**D**)HR(95%CI: 0.519–0.754, *P* = 0.028), ROC curves of STEMI: (**E**)AI(95%CI: 0.528–0.749, *P* = 0.026), ROC curves of NSTEMI: (**F**)combined(95%CI: 0.607–0.819, *P* = 0.001). HR: heart rate; AI: arteriosclerosis index

**Fig. 4:**
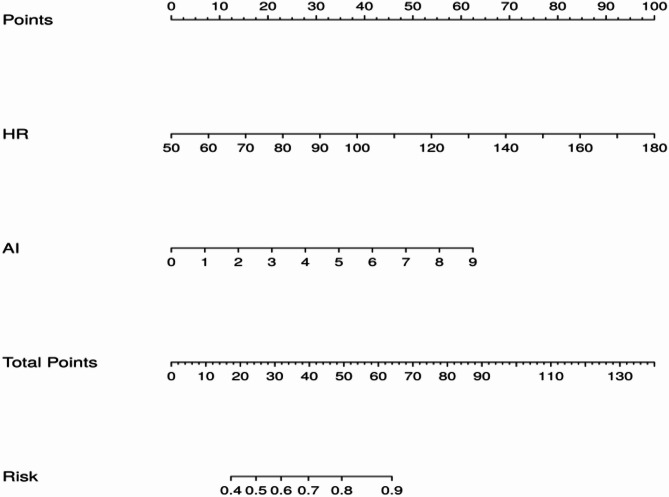
Nomogram prediction model for LAD in ACS patients

To evaluate the efficacy of HR, AI, and a combination of the two in predicting the degree of LCA lesions in patients with ACS, we analyzed the sensitivity, specificity, optimal cut-off value, and area under the curve of the model using ROC curves. As shown in the Table 3; Fig. 3, in ACS patients with concomitant RCA lesions, the optimal cut-off value for HR to predict the degree of LCA lesions in patients with ACS was 76, the sensitivity was 59.1%, the specificity was 69.4%, and the area under the ROC curve was 0.70 (95%CI: 0.644–0.756, *P* < 0.001). The optimal cut-off value of AI for predicting the degree of LCA lesions in ACS patients was 3.3, the sensitivity was 71.6%, the specificity was 47.1%, and the area under the ROC curve was 0.59 (95%CI: 0.533–0.656, *P* = 0.031). As for HR combined with AI for predicting the degree of LCA lesion in ACS patients, the sensitivity was 74.2%, the specificity was 60.0%, and the area under the ROC curvature was 0.72 (95%CI: 0.667–0.775, *P* < 0.001). In ACS patients with isolated LCA lesions, both AI(AUC = 0.64, 95%CI: 0.519–0.754, *P* = 0.028) and HR(AUC = 0.64, 95%CI: 0.528–0.749, *P* = 0.026) retained predictive value for the severity of LCA lesions, with their combination(AUC = 0.71, 95%CI: 0.607–0.819 *P* = 0.001) demonstrating superior predictive performance. The results showed that HR and AI had similar ROC curves in predicting the degree of LCA lesions in ACS, but the predictive value of the two combinations was greater than that of the two groups alone, and the difference was statistically significant (*P* < 0.05). Additionally, we performed separate receiver operating characteristic (ROC) analyses for STEMI and NSTEMI subgroups within the ACS population. The comparable AUC values between these groups suggest equivalent predictive performance across ACS subtypes. Additionally, we derived risk prediction scores by transforming the values of all predictive variables in the model, with the cumulative score reflecting an individual’s probability of risk or survival (Fig. 4). Meanwhile, internal validation was conducted focusing exclusively on ACS patients presenting with isolated LAD(Supplementary Figure-1). The precision-recall curves and calibration plots for the validation set were generated to evaluate the prediction model, evaluation of precision-recall performance yielded an AP score of 0.882 in the training set and 0.857 in the validation set. The test set demonstrated a comparatively reduced AUC value(AUC = 0.54). Finally, we evaluated both the net reclassification improvement (NRI) (Table 4)and integrated discrimination improvement (IDI) (Table 5) indices for the single-variable versus combined-variable models. The results demonstrated statistically significant improvements for the combined model, with both NRI and IDI values exceeding 0 (all *P* < 0.05). These findings quantitatively confirm that the combined-variable model provides superior predictive capacity compared to single-variable model. Finally,

**Table 3:** Predictive power of HR and AI for LCA lesions in patients with ACS and its subgroups

	Category	Variable	AUC	SE	*P*-value	95%CI	Cut-off value	Sensitivity	Specificity	Youden index
LCA (with or without RCA lesions)	ACS	HR	0.70	0.029	< 0.001	0.644–0.756	76	0.591	0.694	0.285
		AI	0.59	0.031	0.003	0.533–0.656	3.3	0.716	0.471	0.187
		Combined	0.72	0.028	< 0.001	0.667–0.775	0.624	0.758	0.579	0.337
	STEMI	HR	0.69	0.035	< 0.001	0.622–0.760	76	0.595	0.676	0.271
		AI	0.59	0.04	0.024	0.510–0.668	2.87	0.841	0.351	0.192
		Combined	0.72	0.034	< 0.001	0.648–0.782	0.669	0.774	0.568	0.342
	NSTEMI	HR	0.73	0.053	< 0.001	0.627–0.836	69	0.836	0.553	0.389
		AI	0.62	0.056	0.048	0.506–0.727	3.48	0.642	0.632	0.274
		Combined	0.74	0.051	< 0.001	0.643–0.842	0.447	0.940	0.447	0.387
LCA (without RCA lesions)	ACS	HR	0.64	0.06	0.028	0.519–0.754	78	0.568	0.643	0.211
		AI	0.64	0.056	0.026	0.528–0.749	4.09	0.337	0.929	0.266
		Combined	0.71	0.054	0.001	0.607–0.819	0.795	0.547	0.786	0.333
	STEMI	HR	0.52	0.081	0.811	0.361–0.678	81	0.437	0.467	0.096
		AI	0.64	0.079	0.092	0.484–0.794	3.82	0.380	0.867	0.247
		Combined	0.66	0.077	0.055	0.508–0.808	0.818	0.634	0.667	0.301
	NSTEMI	HR	0.75	0.09	0.042	0.567–0.922	76	0.565	0.875	0.44
		AI	0.67	0.097	0.149	0.484–0.864	3.89	0.522	0.875	0.397
		Combined	0.82	0.079	0.008	0.665–0.976	0.813	0.609	0.875	0.484

ACS: Acute coronary syndromes; AI: arteriosclerosis index; HR: heart rate; STEMI: ST-segment elevation myocardial infarction; NSTEMI: non-ST-segment elevation myocardial infarction; AUC: area under Curve; SE: standard error; CI: confidence interval; RCA: right coronary artery; LCA: left coronary artery

**Table 4:** The predictive performance of the index using NRI

	Index	Estimate	SE	Lower	Upper	*P*-value
LCA (with or without RCA lesions)	NRI	0.74931129	0.10219142	0.577594939	0.88532721	< 0.001
	NRI+	0.03030303	0.04163199	−0.030888031	0.06299213	0.467
	NRI-	0.71900826	0.07905431	0.568090909	0.85479537	< 0.001
	Pr(Up|class)	0.03030303	0.01555138	0.007889823	0.07090875	–
	Pr(Down|class)	0.00000000	0.03759155	0.000000000	0.05747211	–
	Pr(Down|Ctrl)	0.71900826	0.07334423	0.576872488	0.85773810	–
	Pr(UP|Ctrl)	0.00000000	0.01494010	0.000000000	0.02459182	–
LCA (without RCA lesions)	NRI	0.51052632	0.17022131	0.1682900	0.82539770	0.003
	NRI+	0.01052632	0.05612658	−0.1629171	0.05586767	0.851
	NRI-	0.50000000	0.14742009	0.2357143	0.81365741	0.001
	Pr(Up|class)	0.01052632	0.01976683	0.0000000	0.06002401	–
	Pr(Down|class)	0.00000000	0.05076147	0.0000000	0.17708333	–
	Pr(Down|Ctrl)	0.53571429	0.13032866	0.3162338	0.81481481	–
	Pr(UP|Ctrl)	0.03571429	0.03802979	0.0000000	0.12500000	–

NRI: net reclassification improvement; SE: standard error; RCA: right coronary artery; LCA: left coronary artery

**Table 5:** The predictive performance of the index using IDI

	Index	Hazard ratio	Lower	Upper	*P*-value
LCA lesions (with or without RCA artery lesions)	IDI	0.6403	0.5856	0.6950	< 0.001
LCA lesions (without RCA lesions)	IDI	0.4904	0.3687	0.6122	< 0.001

IDI: integrated discrimination improvement; LCA: left coronary artery; RCA: right coronary artery

## Discussion

Previous studies have demonstrated that in ACS patients, particularly those with acute myocardial infarction, LCA lesions or stenoses are significantly more likely to serve as culprit lesions compared to RCA involvement. The INTERHEART Study pointed out that left anterior descending (LAD) is the most common culprit vessel in acute myocardial infarction (accounting for more than 40%) [21]. In the KAMIR study, LCA lesions accounted for 49.1% of acute myocardial infarction (AMI) patients, and RCA lesions accounted for 34.3% [22]. In the China PEACE Study, the left anterior descending artery was the most frequently involved vessel in Chinese AMI patients (48.2%), and the RCA accounted for 37.6% [23]. The 2023 ESC ACS Management Guidelines also mentioned that the LCA system (especially LAD) is the most common culprit vessel in ACS [24]. Therefore, early prediction of LCA lesions is clinically imperative. In recent years, most studies on the impact of lipids on cardiovascular diseases have focused on atherogenic index of plasma (AIP) (calculated as log10 (TG/HDL-C)). Multiple studies have shown that AIP levels are significantly correlated with the degree of coronary artery lesions [25–27], but these studies have not separately studied LCA or RCA. However, in our clinical work, it is indeed rare to encounter ACS patients with only LCA lesions. Most patients have multi-vessel coronary artery lesions, with varying degrees of stenosis, but not necessarily all being culprit vessels. Epidemiological studies have shown a linear correlation between HR and cardiovascular disease risk. The Framingham cardiac study suggests that mortality from cardiovascular disease is inversely correlated with resting HR levels [28], and a population study of hypertensive patients with coronary artery disease has reached similar conclusions [29]. In a two-year follow-up of 579 surviving patients with acute myocardial infarction, Copie et al. found that the 24-hour mean HR was a better predictor of the risk of all-cause mortality, cardiovascular mortality, and sudden death than left ventricular ejection fraction [30]. The resting HR is an important indicator of myocardial oxygen demand and oxygen consumption, and the decrease in HR indicates a decrease in cardiac workload, which is beneficial to the prognosis of cardiovascular disease.

In this study, we included a total of 385 patients with LCA lesions in ACS, among whom 262 patients had RCA lesions. There were 53 patients with a higher right coronary GS score than the left coronary score, 123 patients(31.95%) had isolated LCA lesions, and 101 patients (26.23%) had the RCA as the culprit vessel. There were 239 patients (62.08%) with the LCA as the culprit vessel, which is consistent with the high incidence of LCA lesions as culprit vessels in the above studies. In recent years, studies on the correlation between AI and the degree of coronary artery lesions and its predictive value are rare. Our study starts from AI and only selects a single coronary artery (LCA) for research. This study found that HR and AI were both positively correlated with the Gensini score of the LCA. Therefore, we believe that HR and AI have certain predictive value for the degree of LCA lesions, and the combined predictive value of the two for the degree of LCA lesions may be higher. In our prediction of the degree of LCA lesions(with concomitant RCA lesions) by HR, the area under the ROC curve was 0.70, the sensitivity was 59.1%, and the specificity was 69.4%. However, in patients with isolated LCA lesions, the AUC of HR decreased to 0.64. We postulate that this reduction may be attributable to the contribution from RCA lesions, as RCA lesions could potentially impair sinoatrial node function and consequently affect HR. In the prediction of the degree of LCA lesions by AI, the area under the ROC curve was 0.59, the sensitivity was 71.6%, and the specificity was 47.1%. We can see that the sensitivity of HR in predicting LCA lesions is relatively low, the AUC value and the specificity of AI in predicting LCA lesions is relatively low. We attribute this confounding effect to the inclusion of patients with RCA lesions, as the AI demonstrated improved predictive performance (AUC = 0.64) in ACS patients with isolated LCA lesions. We hypothesize that the predictive power would be further enhanced with a larger sample size of this specific patient subgroup.When we combine HR and AI to predict the degree of LCA lesions(with RCA lesions), the area under the ROC curve is 0.72, the sensitivity is 74.2%, and the specificity is 60%. Similarly, in ACS patients with isolated LCA lesions, AUC is 0.71, the sensitivity is 54.7%, and the specificity is 78.6%. The sensitivity and specificity are still not high enough. We consider that first, the sample size of patients included in this study is relatively small. Secondly, the distribution of some baseline indicators (HR, TC, TG, LDL-C, Lesions of RCA, AI, Gensini score (LCA)) is unbalanced (*P* < 0.05). Of course, this imbalance may also be caused by the low sample size. Our internal validation revealed a decreased AUC in the test set, suggesting possible model overfitting that might be attributable to limited training sample size.

## Conclusions

This study identified that the combination of HR and arterial stiffness index demonstrates predictive value for assessing LCA lesion severity in ACS patients. We aim to utilize these readily obtainable clinical parameters (initial admission HR and lipid profiles) to facilitate early prognosis prediction, thereby enabling clinicians to provide more comprehensive and precise therapeutic interventions during the critical early phase of ACS, with the ultimate goal of improving clinical outcomes. The universal availability of HR and arterial stiffness measurements across primary care clinics, community hospitals, and tertiary medical centers ensures broad applicability of our findings.

### Limitations

This study is a single-center retrospective study, which has the following several limitations. First, a total of 385 patients were included in this study, with a relatively small sample size. Secondly, The distribution of some baseline indicators was unbalanced (HR, TC, TG, LDL-C, RCA lesions, AI, Gensini score). Of course, this imbalance might also be caused by the low sample size. Thirdly, during internal validation, a relatively lower AUC in the test set, potentially indicating overfitting. Finally, the combined model of AI and HR demonstrated moderate predictive accuracy, suggesting potential for further optimization to enhance its clinical utility.

## Electronic supplementary material

Supplementary Material 1

## Data Availability

No datasets were generated or analysed during the current study.

## Abbreviations

ACS: Acute coronary syndromes
AF: Atrial fibrillation
AI: Arteriosclerosis index
AIP: Atherogenic index of plasma
AMI: Acute myocardial infarction
AP: Average precision
AUC: Area under Curve
BMI: Body mass index
CI: Confidence interval
CKD: Chronic kidney disease
DBP: Diastolic blood pressure
eGFR: Estimated glomerular filtration rate
FFR: Fractional flow reserve
HDL-C: High density lipoprotein-cholesterol
HR: Heart rate
IDI: Integrated discrimination improvement
LAD: Left anterior descending
LCA: Left coronary artery
LDL-C: Low-density lipoprotein cholesterol
NRI: Net reclassification improvement
NSTEMI: Non-ST-segment elevation myocardial infarction
OR: Odds ratio
RCA: Right coronary artery
ROC: Receiver operating characteristic curve
SBP: Systolic blood pressure
SE: Standard error
STEMI: ST-segment elevation myocardial infarction
TC: Total cholesterol
TG: Triglyceride
