# Supplementary material for: Prediction value of heart rate combined with arteriosclerosis index for left coronary artery lesion in patients with ACS
Source: BMC Cardiovasc Disord. 2025 Aug 2;25:569. doi: 10.1186/s12872-025-05063-2 (PMC12317464; doi:10.1186/s12872-025-05063-2)

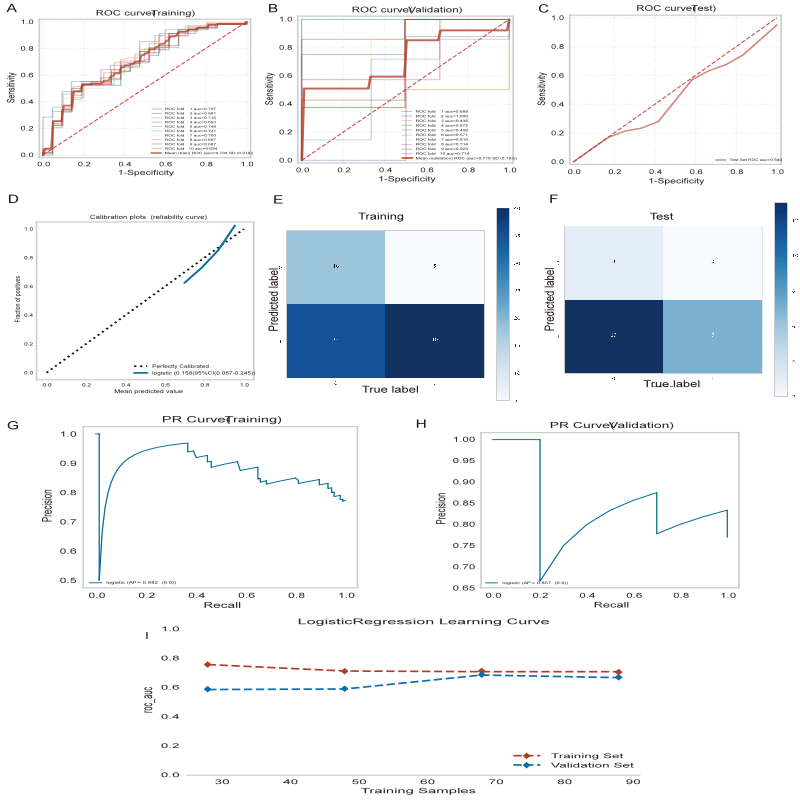


Supplementary Figure-1. Internal validation was conducted focusing exclusively on ACS patients presenting with isolated LAD. (A,B,C)ROC curves for the training(Mean ROC=0.704), validation(Mean ROC=0.718) and test(AUC=0.540) set.(D) Calibration curve analysis. (E)Training set confusion matrix for the optimal model. (F)Test set confusion matrix for the optimal model. (G)Precision-recall for training set(AP=0.882). (H)Precision-recall for validation set(AP=0.857). (I)Logistic regression learning cure between training set and test set。


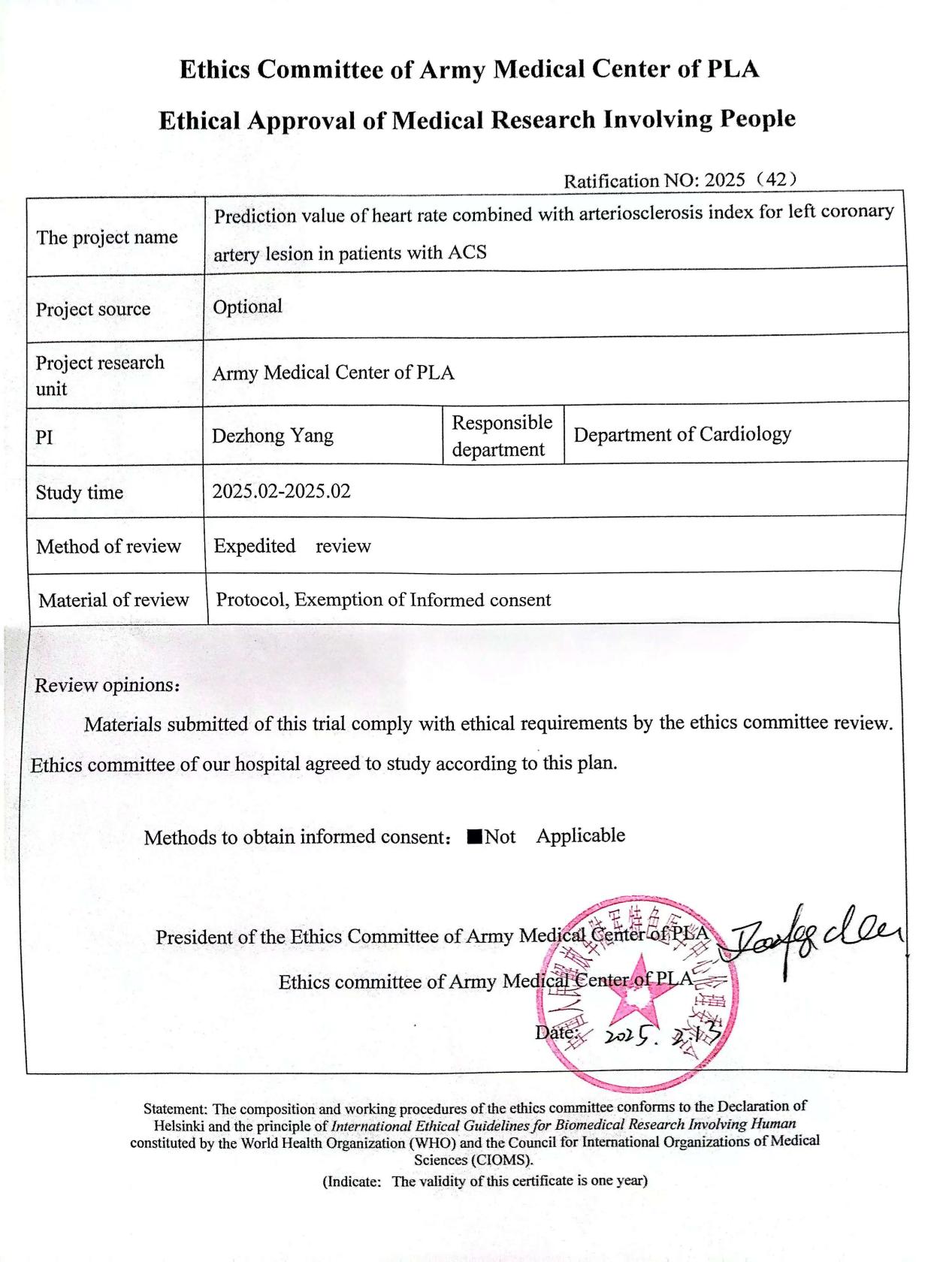

Supplement: Supplementary file 1 — Supplementary Material 1 [file 12872_2025_5063_MOESM1_ESM.docx]
